# Polaritonic Rabi and Josephson Oscillations

**DOI:** 10.1038/srep28930

**Published:** 2016-07-25

**Authors:** Amir Rahmani, Fabrice P. Laussy

**Affiliations:** 1 Physics Department, Yazd University, P.O. Box 89195-741, Yazd, Iran; 2 Russian Quantum Center, Novaya 100, 143025 Skolkovo, Moscow Region, Russia; 3 Condensed Matter Physics Center (IFIMAC), Universidad Autónoma de Madrid, E-28049, Spain

## Abstract

The dynamics of coupled condensates is a wide-encompassing problem with relevance to superconductors, BECs in traps, superfluids, etc. Here, we provide a unified picture of this fundamental problem that includes i) detuning of the free energies, ii) different self-interaction strengths and iii) finite lifetime of the modes. At such, this is particularly relevant for the dynamics of polaritons, both for their internal dynamics between their light and matter constituents, as well as for the more conventional dynamics of two spatially separated condensates. Polaritons are short-lived, interact only through their material fraction and are easily detuned. At such, they bring several variations to their atomic counterpart. We show that the combination of these parameters results in important twists to the phenomenology of the Josephson effect, such as the behaviour of the relative phase (running or oscillating) or the occurence of self-trapping. We undertake a comprehensive stability analysis of the fixed points on a normalized Bloch sphere, that allows us to provide a generalized criterion to identify the Rabi and Josephson regimes in presence of detuning and decay.

Some macroscopic quantum systems such as a superconductor can be described by an order parameter, that reduces the dynamics of a complex object to a simple complex number^1^. The question of what happens with the phases of two superconductors put in contact through an insulating barrier led Josephson to predict with elementary equations that a supercurrent should flow between them, driven by their phase difference^2^. The phenomenon was quickly observed^3^ and became emblematic of broken symmetries and quantum effects at the macroscopic scale. It was soon speculated that a similar phenomenology should be observed with other degenerated quantum phases, such as superfluids or Bose–Einstein condensates (BECs), even before the latter were experimentally realized^4^. The role of the phase as the driving agent of quantum fluids was brought to the fore by Anderson^5^ who identified “phase slippage” as a source of dissipation^6^. The first transposition of this physics to the case of BECs was considering non-interacting particles^4^ and the role of the phase difference as the source of the superflow was the focus of attention. The question of the phase of macroscopically degenerate quantum states remained anchored in the phenomenon but also took a separate route of its own^7^,^8^,^9^, that is still actively investigated to this day^10^,^11^.

The Josephson effect itself, on the other hand, was put on its theoretical foothold by Leggett who defines it as the dynamics of *N* bosons “restricted to occupy the same two-dimensional single particle Hilbert space”^12^. Leggett introduced three regimes for such systems depending on the relationship between tunelling and interactions, namely the Rabi (non-interacting), Josephson (weakly-interacting) and Fock (strongly-interacting) regimes^13^. “Tunneling” refers to linear coupling between the condensates (quadratic in operators) while “interactions” refer to a nonlinear self-particle quartic term. In this sense, Josephson’s physics is a limiting case of the Bose–Hubbard model^14^, although the name retained a strong bond with superconductors^15^, possibly due to the important applications it found as a quantum interference device^16^,^17^ or merely for historical reasons (the Josephson–Bardeen debate on the existence of the effect is one highlight of scientific controversies^18^). To mark this distinction of pure-boson implementations of the Josephson dynamics from those involving Cooper pairs, one speaks of “Bosonic Josephson junctions” for the former case (BJJ)^19^. This typically relates to condensates trapped in two wells, but due to its fundamental and universal character as formulated by Leggett, numerous other platforms exhibit the effect. A pioneering report came from superfluids^20^. For proper BECs, before a direct observation between two spatially separated condensates, a so-called “internal” Josephson effect was deemed “more promising” by involving different hyperfine Zeeman states of alkali gases^13^ (the Josephson oscillation in a single junction of BECs was observed in 2005^21^). In this text, we consider another platform that can host Bose condensates: microcavity polaritons^22^. These systems having demonstrated Bose–Einstein condensation^23^ and superfluid behaviour^24^, are natural candidates to implement the Josephson physics of coupled condensates, furthermore, in strongly out-of-equilibrium open systems. Several theoretical proposals have been made^25^,^26^,^27^, followed by experimental observations reported in both the linear (Rabi)^28^ and nonlinear (self-trapping)^29^ regime. The polariton implementation of Josephson effects is increasingly investigated^30^,^31^,^32^,^33^,^34^,^35^,^36^,^37^,^38^. Recently, it was observed that polaritons are predisposed for Josephson physics from the very nature of their light-matter composition^39^, exhibiting innately the internal type of such Josephson dynamics where the exchange is not between two spatially separated condensates but between the two internal degrees of freedom that make up the polariton, namely, its exciton and photon components. This is an adequate picture, since condensates of polaritons are also condensates of photons and excitons^40^. The Rabi coupling acts as the tunneling and interactions are then for the excitonic component only, bringing a variation on the atomic counterpart in space, and detuning of the free energies between the modes act as the external potential, so the analogy is essentially complete. Since the polariton BEC order parameter needs not vary in space (but see refs 41,42), both frameworks—order parameters of coupled condensates and dissipative quantum optics—are tightly related and each sheds a new light on the other. The relations between quantum optics and Josephson dynamics have been scarce but far-reaching, as illustrated by the Josephson quantum optical interferometer^43^. They also fill-in some conceptual gaps for both aspects. In particular, the phase dynamics between the modes has been essentially ignored in the quantum optical viewpoint of light-matter coupling^44^,^45^,^46^, while the general case with an interplay of detuning, different on-site interactions and a finite lifetime has not been considered for Josephson oscillations despite the relevance of all these aspects, and not only for polaritonic systems. We show how such a wider picture blurs the line between Rabi and Josephson dynamics, or, rephrased more positively, provides an elegant and natural physical picture that brings the two regimes closer together. Most importantly, we provide a general criterion to take into account these new parameters, that should be considered to claim the Josephson regime in dissipative systems beyond the simple observation of oscillations or of a running phase. In absence of such a solid criterion, any claim of Josephson physics in system with variations from the atomic paradigm (infinite lifetime, resonance, indistinguishable particles, etc.) has to received critically.

## Theory

The dynamics of the Bosonic Josephson effect has been considered extensively by Raghavan *et al*.^47^ in a form suitable for our discussion, including some considerations of dissipation^48^ (see ref. 19 for a review). We now briefly introduce the main formalism and notations. We refer to the Methods Section for details and will focus on the discussion of the results.

The Bosonic Josephson physics describes the coupling between two weakly-interacting Bose fields, *a* (e.g., left trap/photons, etc.) and *b* (e.g., right trap/excitons, etc.), with possibly different free energies *ε*_*a*,*b*_, ruled by the Hamiltonian *H* = *H*_0_ + *V* where:




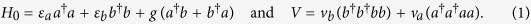




The dynamics is typically described in terms of i) the population imbalance *ρ* ≡ (〈*a*^†^*a*〉 − 〈*b*^†^*b*〉)/2 between the two modes and ii) their relative phase *σ* = arg〈*a*^†^*b*〉 (See Methods and the Supplementary Material for descriptions through alternative variables, in particular for the case of internal Josephson oscillations of polaritons when only the optical field is available). The relative phase is, strictly speaking, *S* ≡ arg(〈*a*〉 − 〈*b*〉) while we define it here as *σ*, the argument of a first-order cross-correlation. This is done for greater generality as it allows us to describe all types of quantum states for coupled harmonic oscillators, including mixed states. For coherent states (describing ideal condensates), *S* = *σ*, and our convention thus causes no loss of generality. Note that such mean-field approximations have been relaxed in recent years and exact (numerical) solutions are now available^49^,^50^ that, interestingly, depart considerably from the established picture, in particular regarding the role of the phase. The two key observables are ruled by the following equations of motion^39^,^47^:




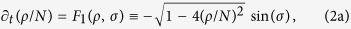







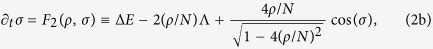




where we introduce the notation *F*_1,2_ for future convenience, *N* ≡ 〈*a*^†^*a*〉 + 〈*b*^†^*b*〉 is the total number of particles and *δ* = (*ε*_*a*_ − *ε*_*b*_)/*g* is the bare modes detuning. Two parameters are paramount to describe the dynamics: an effective detuning Δ*E* and an effective blueshift Λ, defined as:




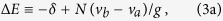







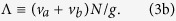




**Figure 1:**
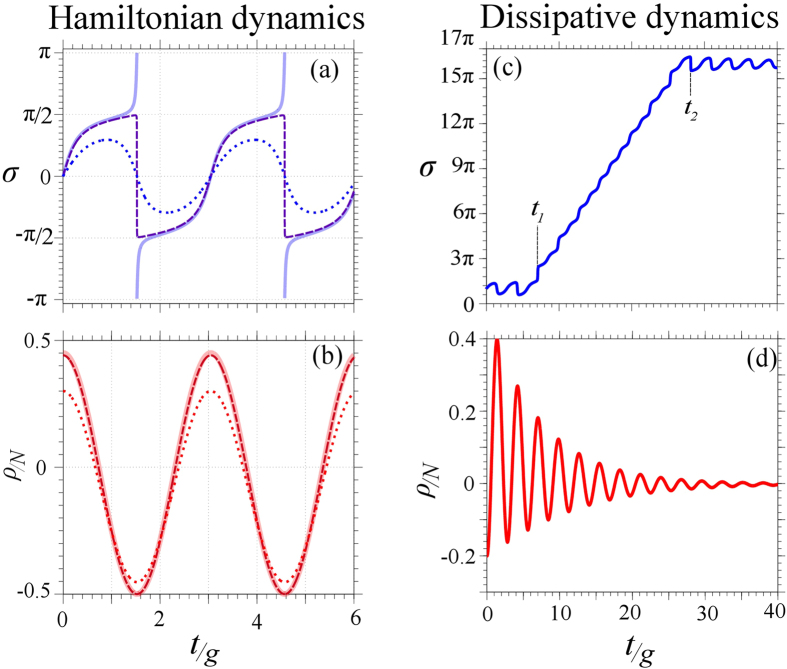
Dynamics of the relative phase σ and population imbalance *ρ* in a variety of scenarios of the pure Rabi regime: (**a**) Transition in the Hamiltonian regime from an oscillating-phase (dotted blue) to a running phase (solid light blue, “not unwrapped” so it is discontinuous). For the oscillating-phase regime with sinusoidal oscillations, parameters are *ρ*_0_ = 0.3 *N*, *σ*_0_ = 0 and *δ* = −0.5. For the oscillating-phase regime with strongly anharmonic oscillations of the phase (dashed purple), parameters are *ρ*_0_ = 0.45 *N*, *σ*_0_ = 0 and *δ* = −0.5. For the running-phase regime (or discontinuous jumps), parameters are: *ρ*_0_ = 0.48 *N*, *σ*_0_ = 0 and *δ* = −0.5. (**b**) Corresponding oscillations in the population, remaining in all cases sinusoidal. (**c**) Relative phase and (**d**) population imbalance in the dissipative regime (with decay but without pumping nor interactions). There is a transition from the oscillating-phase to the running-phase regime at *t*_1_ and back at *t*_2_, with no notable feature in the population imbalance. Parameters: *ρ*_0_ = −0.2 *N*, *σ*_0_ = *π*, *δ* = −1, *γ*_*a*_ = 0.22 *g* and *γ*_*b*_ = 0.02 *g*, in which case *t*_1_ ≈ 6.9 *g* and *t*_2_ ≈ 28 *g*.

Voronova *et al*.^39^ recently reported a peculiar phase dynamics of BJJ when including detuning, even in the linear regime: the phase oscillations are strongly anharmonic and possibly even get in a regime of phase-jumping (or freely running phase if unwrapped). This is noteworthy as reminiscent of the Josephson dynamics, i.e., driven by interactions. Without interactions, oscillations in populations remain harmonic for all detunings, indeed with some renormalization of frequencies and nonzero imbalance, as can be expected from the conventional picture of Rabi coupling out of resonance. Particular cases of this time dynamics for *ρ* and *σ* are shown in Fig. 1. In panel (a), the relative phase *σ* is seen to oscillate between −*π*/2 and *π*/2 (broken lines) or run (solid line, discontinuous in [−*π*, *π*]) depending on the initial condition and detuning, with strongly anharmonic oscillations close to the transition. In all cases, particles transfer harmonically between the two states as oscillations in *ρ* show in panel (b). There follows a rich phase diagram that can be characterized analytically^39^ in the linear or weakly interacting regime. Here it must be stressed again that the same phenomenology that is usually attributed to Josephson dynamics is observed without interactions, that is, in the pure Rabi regime. This calls to reconsider what is meant, precisely, by Josephson and Rabi dynamics. We clarify this point below.

In the quantum-optical mindset, dissipation is an essential part of the dynamics of coupled oscillators^45^,^51^. This is also an ingredient that is crucial to describe short-lived polaritons. To do so, the formalism is upgraded from an Hamiltonian to a Liouvillian description, leading to a master equation for a density matrix *ρ*, which is a standard procedure outlined in the Methods. This introduces the decay rates *γ*_*a*_ and *γ*_*b*_ at which particles from each mode are lost. One example of the dissipative Rabi dynamics is shown in Fig. 1(c,d). Remarkably, one observes a switching in time between the oscillating and running phase regimes. This switching due to the decay also depends on the initial condition as well as detuning. The switching happens if the occupation in one state becomes exactly zero, with all the population residing in the other state, although this is not a necessary condition. When this occurs, the phase of the emptied state becomes ill-defined and so does the relative-phase. Such a change of regime can appear two times, as shown in the figure, a single time or none. In any case, the system always ends up in the regime of oscillating phase, except at resonance where the running mode can last forever. There is therefore only one switching when the initial condition and detuning are such that the dynamics starts in the running-phase mode. These are mere statements of the facts. We will explain the reason for this peculiar behaviour in the following and it will become clear that such an apparently rich phenomenology is in fact trivial and bears no connection to Rabi and Josephson dynamics.

The Rabi/Josephson dynamics is put in full view geometrically in the (*ρ*, 〈*a*^†^*b*〉) space. Since 〈*a*^†^*b*〉 is complex, the space is three-dimensional and the equation of motion can be found in a simple form (see Methods):




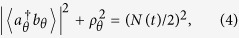




where *θ* is the *mixing angle* between exciton and photons, i.e., *a*_*θ*_ and *b*_*θ*_ are the annihilation operators for the polaritons. In the (*ρ*, 〈*a*^†^*b*〉) space, the trajectory is therefore simply that of “circles on a sphere”. In non-Hamiltonian cases, the radius changes in time but solutions remain equally simple if kept on a normalized sphere, that is a counterpart of the Bloch sphere, that describes the dynamics of a two-level system.

## Hamiltonian Regime

### Dynamics

**Figure 2:**
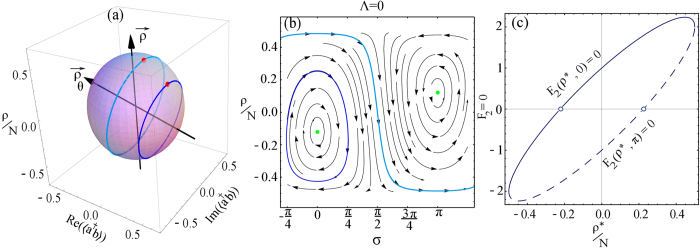
(**a**) The dynamics of two coupled condensates is clearly understood on a Bloch sphere. The polariton basis defines an axis 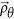 around which the pure Rabi dynamics evolves as a simple circle, whose distance from the center is determined by the quantum state, with polaritons at the poles and the full-amplitude Rabi oscillations between the dressed states as the equator. At resonance, *δ* = 0, 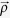 and 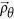 are orthogonal. parameters are the same as Fig. 1. (**b**) Projection of the dynamics of the two cases in panel (a) on the (*ρ*, *σ*) space, superimposed on the streamlines of the dynamical system. There are two centers, displayed as green dots, located at *σ* = 0 and *σ* = *π*. (**c**) The fixed points are solutions of *F*_2_(ρ^*^, σ^*^) = 0, that is, the intersection of the curve with the *x* axis, indicated by open circles. The solid line corresponds to *σ* = 0 and the dashed one to *σ* = *π*. The detuning was taken as Δ*E* = 0.5.

We first revisit the usual Hamiltonian case with no dissipation, i.e., when *γ*_*a*_ = *γ*_*b*_ = 0 and *N*(*t*) is constant. The pure Rabi regime, when *v*_*a*_ and *v*_*b*_ are zero, admits analytical solution^39^. The perplexing dynamics of the phase is easily understood on the Bloch sphere, as shown in Fig. 2(a), where the Rabi dynamics reduces to an exact circle. This circle is concisely and fully described by its normal axis 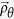 and its distance *ρ*_*θ*_ from the equator in this basis. The latter is given by:




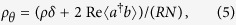




where 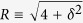. This is a clear result in quantum-optical terms: in the proper basis—of dressed states—the dynamics is that of the free propagation (circular motion) of uncoupled states. This is determined by the 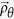 axis, around which the dressed states evolve freely (harmonically) at a distance from the equator that is determined by their state (their content of lower and upper polaritons), leading to a linearly increasing phase. We can now clarify that what determines the dynamics of the phase (oscillating or running) is simply whether the trajectory on the sphere encircles or not the South–North 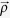 axis defined by the laboratory observables (i.e., of the bare states). In the basis of dressed states, the phase is always running. Bare states on the other hand are the familiar physical objects of the system in which terms it is convenient to think. In the internal BJJ version, they are the exciton and photon modes, and are furthermore those typically observed experimentally (only the photons in most experiments). This laboratory basis is, in the case of optimal strong-coupling, orthogonal to the dressed state basis, with 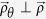, and the circular motion is observed as a sinusoidal oscillation (a circle observed sidewise) in the general case, or even a saw-tooth function when the quantum state maximizes the amplitudes of oscillations by satisfying *ρ*/*N* = ±1/2 (for instance starting with all polaritons in one mode at *t* = 0). An example is shown in Fig. 2, namely, as initial conditions: a 50-50 (light blue) superposition of *θ*-eigenstates and another ratio (in blue), leading to a smaller circle, both normal to the 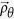 axis. As observed in the exciton-photon basis, their *ρ* and *σ* dynamics is distorted. There is no such distortion for the population imbalance, since the circular motion from any circle on the sphere projected on any normal axis still results in a sine function. However the relative phase is defined by the phase of the vector that joins the center of the sphere and the circle itself. If the circle lies outside the axis, the phase can remain always unequivocally defined in a 2*π* interval, leading to oscillations as the trajectory reaches an apex on the sphere and turns back. This is the situation of the blue circle in Fig. 2(a). In the other case where the circle goes round the axis, there is no turning point and the phase increases forever. This is the situation of the light blue circle in Fig. 2(a). It is clear, then, that the dynamics of the phase has no deep meaning of driving a flow of particles. Instead, it pertains to a choice of basis. The oscillating phase regime corresponds to a case where the basis of observables is too far apart from that which is natural for the system (eigenstates) and the tilt between their axes is so large that the phase is distorted into a qualitative different behaviour of oscillations instead of a linear drift. When the circle lies outside but close to the observable axis, the oscillations are highly distorted. In contrast, the running phase regime is that where the system is described by observables close to the dressed states of the system.

The rationale of Leggett in distinguishing between a Rabi and a Josephson regime was to set apart the cases where tunneling (in our case, the Rabi coupling *g*) dominates over the nonlinearity (*v*, the common self-interaction *v*_*a*_ = *v*_*b*_). At resonance and for equal interaction on both sites, the criterion is to compare 2*vN*/*g* to unity. This is indeed correct but, even in absence of dissipation, is restricted to resonance and equal nonlinearities. We proceed to provide the general result to set apart the Rabi and Josephson regimes in presence of detuning, which is required in general even for Hamiltonian systems since detuning alone may fake a Josephson-looking dynamics in non-interacting systems.

### Classification of fixed points

**Figure 3:**
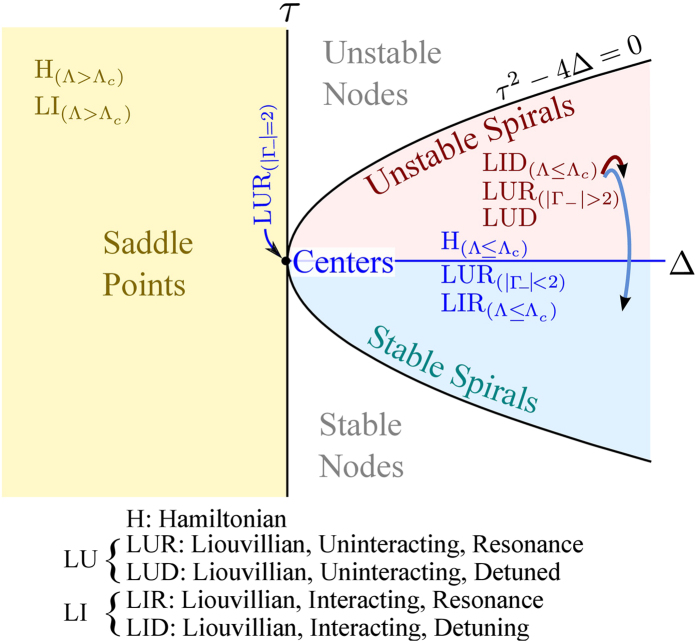
Classification of the fixed points of the dissipative Bosonic Josephson Junction. The axes are the functions Δ ≡ *λ*_1_*λ*_2_ and *τ* ≡ *λ*_1_ + *λ*_2_ of the Jacobian’s eigenvalues, cf. Eq. (6). Our terminology is spelled out at the bottom of the figure, with (at most) three letters to label each case: first letter is either H (Hamiltonian) or L (Liouvillian) for the cases without or with decay, respectively. Second letter is U (uninteracting) or I (interacting) for the cases without or with self-interactions, respectively. Third letter is R (resonance) or D (detuned) for the cases *δ* = 0 or *δ* ≠ 0, respectively. Further criteria are specified as subscripts. For instance, 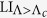 are dissipative systems with interactions with both zero and nonzero detuning such that Λ > Λ_*c*_, in which case these systems have fixed points with saddle instability. The fact that Λ > Λ_*c*_ is equivalent to the existence of a saddle fixed point allows to use the latter as a necessary and sufficient criterion for the Josephson regime. The Nodes area, separated from the Spirals by *τ*^2^ − 4Δ = 0, are not accesible. The purple and blue arrows for the points 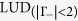 and 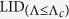 mean that these points belong to both the Unstable and Stable Spirals regions. All the cases shown here are for Δ*E* = 0.

As we are dealing with a dynamical system, the standard procedure to classify the possible trajectories is a stability analysis around the fixed points. In the BJJ, the fixed points ρ^*^ and σ^*^ are by definition the solutions *F*_*i*_(ρ^*^, σ^*^) = 0 for *i* = 1, 2 (cf. Eq. (2)). There are two possible solutions for the phase, σ^*^ = 0 and σ^*^ = *π* (modulo 2*π*, so that σ^*^ = −*π* is also a solution in a closed 2*π* interval). Solving for the other variable, we exhaust the possible fixed points. Their stability is determined by the eigenvalues *λ*_*i*_ of the Jacobian Matrix:




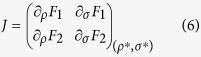




and the type can be mapped on a diagram with axes Δ ≡ *λ*_1_*λ*_2_ and *τ* ≡ *λ*_1_ + *λ*_2_^52^, that is shown in Fig. 3.

#### Non-interacting case

First, in the non-interacting case, the system admits simple closed-form solutions:




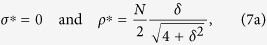







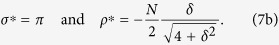




As is clear on physical grounds, detuning can produce a state with a large population imbalance, which can bear resemblance to macroscopic quantum self-trapping even in absence of interaction. Using the definition of Raghavan *et al*.^47^ that the system is macroscopically self-trapped when its total energy balances the coupling strength, we can find a critical detuning that satisfies this condition in absence of interactions, namely:




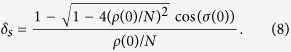




The examples of this dynamics on the Bloch sphere in the noninteracting case shown in Fig. 2(a) are projected in the (*ρ*, *σ*) space in Fig. 2(b), with the two fixed points at *σ* = 0 and *σ* = *π* marked by (green) points. The two orbits show the running and oscillatory phases surrounding these fixed points without being attracted nor repelled by them. In the terminology of dynamical systems, this corresponds to fixed points that are neutrally stable. Geometrically, the fixed points are the intersections with the *x*-axis of the curves shown in Fig. 2(c) (zeros of *F*_2_). For the stability that follows from Eq. (6), we find 
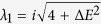
 and 
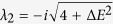
, implying that Δ > 0 and *τ* = 0. As a consequence, the two fixed points in the Rabi regime are centers, i.e., they are stable and every near-enough trajectory is closed^52^. These are the 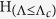 points in Fig. 3 with Λ = 0.

#### Interacting case

**Figure 4:**
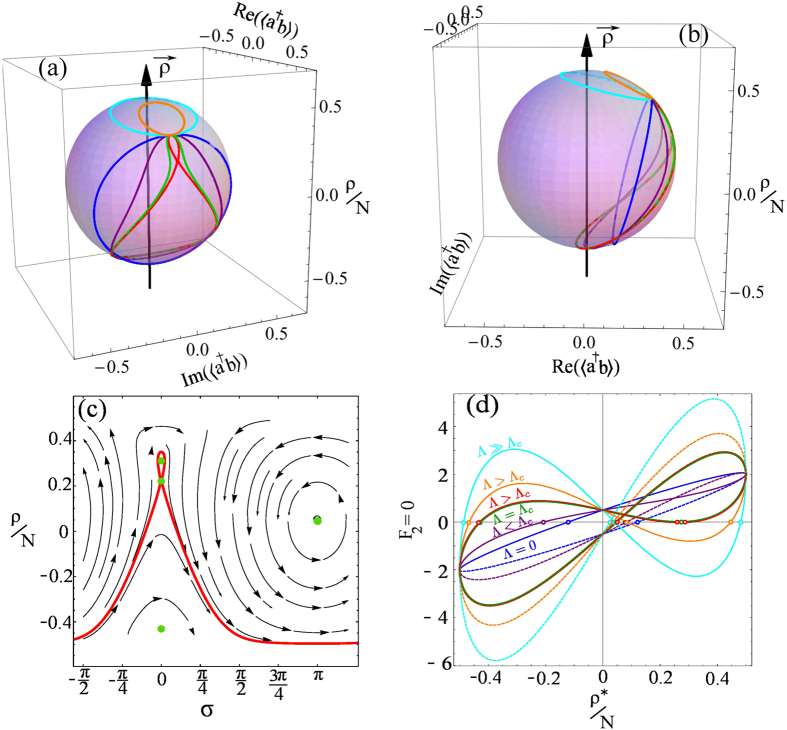
(**a**) Transition from Rabi to Josephson regime. The Blue circle is the pure Rabi (no interaction) regime. Purple is a Rabi-like interacting case, with 0 < Λ < Λ_*c*_, green is the transition case when Λ = Λ_*c*_, red is a Josephson case with Λ slightly over Λ_*c*_, orange and cyan are Josephson cases well above Λ_*c*_. (**b**) Same as (a) but as a side view of the trajectories to show the cases that do encircle or not the 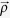 axis, corresponding to oscillatory and running relative phase, respectively. (**c**) Phase-space trajectory of the dynamics in the Josephson regime with a saddle point at *σ* = 0 out of the four fixed points. Each Λ yields its own phase-space vector field, in which a trajectory is followed depending on the initial condition. (**d**) Roots of *F*_2_ = 0, that identify the fixed point in the population imbalance for the relative phase *σ* = 0 mod 2*π* (solid line) and *σ* = *π* mod 2*π* (dashed line). With increasing Λ, the number of roots changes from two (Rabi regime) to four (Josephson regime).

**Figure 5:**
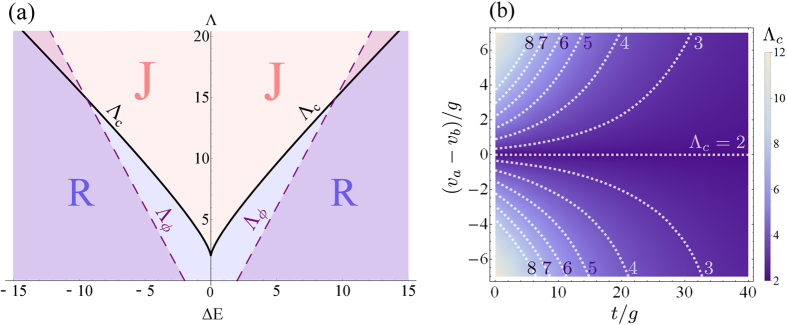
(**a**) Regions of Rabi (R or blue) and Josephson (J or red) regime as a function of Λ and Δ*E*. The frontier, Λ_*c*_ (solid black line) is given by Eq. (10) and provides the general criterion for the Josephson regime in presence of detuning. The frontier denoted by Λ_*ϕ*_ (dashed line) separates the running-phase regime from the oscillating one (shaded area) for the case (*ρ*_0_ = 0, *σ*_0_ = *π*) (Λ_*ϕ*_ depends on the quantum state and we show here the case of greatest extent for the running phase). There is some degree of correlation between running phase and Josephson dynamics but neither implies the other. (**b**) When the interactions of the two condensates are not equal, *v*_*a*_ ≠ *v*_*b*_, the critical Λ_*c*_ becomes time-dependent, as shown here as a density plot for the case of zero detuning. The smallest value is Λ_*c*_ = 2 which is the textbook value for the Josephson regime in the case of equal interactions, at resonance and without dissipation, as recovered here for this particular case. Variations result in an increase of Λ_*c*_, that decays with time to tend towards this fundamental value.

The general interacting case has fixed points solutions in implicit form:




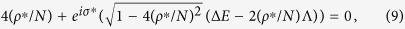




also for σ^*^ ∈ {0, *π*}. Solutions also exist in closed-form but are too bulky to give here. The geometrical solution is, in this case, convenient. It is shown in Fig. 4(d) for various values of Λ. From the shape of the curve, one can see that there are two or four fixed points, and this is the criterion one can unambiguously use to define the Rabi and Josephson regimes, respectively. This can be quantified by studying the order of the discriminant of Eq. (9), yielding the Josephson regime when it is higher than quadratic in *ρ*. This leads us to one important result of this text: the generalized criterion for Josephson dynamics. The critical parameter that separates the Rabi from Josephson regimes in the mean-field approximation is thus:




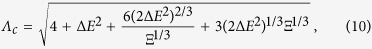




where Ξ ≡ 4 + Δ*E*^2^ + |4 − Δ*E*^2^|. In the literature, the typical configuration reduces to Δ*E* = 0 and yields simply Λ_*c*_ = 2. The diagram in Fig. 5 shows the regions of Rabi (R or blue) and Josephson (J or red) separated by the Λ_*c*_ frontier (black solid line) according to this general criterion. One expects the Josephson regime to occur with increasing effective interaction (Λ). However, this is strongly countered by detuning Δ*E*, that tends to maintain the Rabi regime with a steep increase of the threshold, that is doubled for a detuning of one time the coupling strength only. In highly detuned conditions, the Rabi regime predominates, even with large values of Λ. Note that by “detuning” we mean the effective detuning given by Eq. (3a), that can be considerable for distinguishable particles for which *v*_*a*_ ≠ *v*_*b*_, since it involves the total number of particles.

The fixed points analysis is conveniently done for each value of the phase separately. The σ^*^ = 0 solution yields eigenvalues 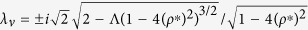 (*ν* = 1, 2), that imply *τ* = 0 and, as far as Λ ≤ Λ_*c*_, Δ > 0 meaning that the fixed points remain centers (these are the 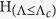 points with nonzero Λ in Fig. 3). However for Λ > Λ_*c*_, one fixed point falls in the region Δ < 0 and becomes a saddle point (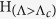). For σ^*^ = *π*, the eigenvalues read 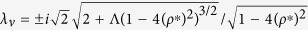 (*ν* = 1, 2) which, for all the values of Λ, results in Δ > 0, meaning that all fixed points around σ^*^ = *π* remain center points (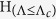), regardless of the strength of the interaction. The existence of one saddle point is thus a robust criterion to identify the Josephson regime in presence of detuning. On Fig. 3 is also superimposed as a shaded area the region of oscillating phase for the case *ρ*_0_ = 0 and *σ*_0_ = *π* (each initial condition yields its own boundary) separated from the region of running phase by the dashed purple line Λ_*ϕ*_. While there is a correlation between the running phase and the Josephson regime, one neither implies nor is implied by the other.

Examples of orbits on the Bloch sphere in the Hamiltonian regime are shown in Fig. 4(a), starting with the blue circle that corresponds to the pure Rabi regime (Λ = 0). With increasing interactions, orbits take the shape of the green trace, that is the frontier between the Rabi and Josephson regimes. Increasing Λ slightly above the critical value, the saddle point appears, corresponding to the Josephson regime. The same orbits are also shown in a side view of the sphere in Fig. 4(b), allowing to see their enclosing or not of the 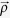 axis and, correspondingly, the running or oscillatory-regime of the relative phase.

## Out-of-Equilibrium (Liouvillian) Regime

We now consider the out-of-equilibrium dynamics, here with decay of the bare states only (we briefly discuss in the Supplementary Material the more complex situation with decay of the dressed states and with pumping).

### Dynamics

**Figure 6:**
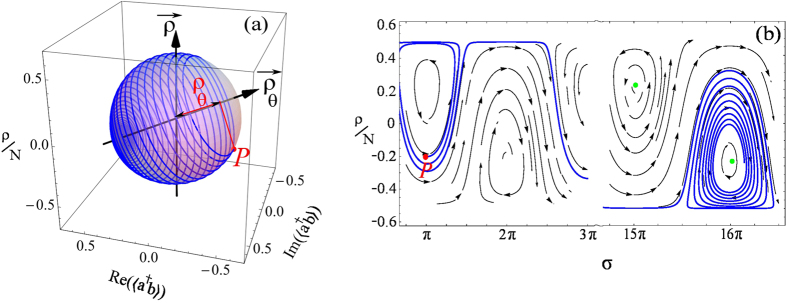
(**a**) Example of the dynamics in the pure Rabi regime (Λ = 0) in a dissipative system. The orbit is an helix on the normalized Bloch sphere (Paria sphere), that starts from the point *P* set by the initial condition and tends toward a steady point on the *ρ*_*θ*_ axes, that remains well defined thanks to the normalization despite the steady state being the vacuum. Parameters are the same as in Fig. 1(h,i). (**b**) Projection of the dynamics on the (*ρ*, *σ*) space, superimposed on the streamlines of the dynamical system. As compared to the Hamiltonian case, the fixed points (displayed as green points) are shifted. Starting from *σ*_0_ = *π*, the spiral gets farther from *π*, then drifts as the system enters in the running-phase regime, and ultimately gets absorbed by the fixed point near 16*π*. The left spiral is unstable, while the right one is stable.

We upgrade the Hamiltonian (H) case to include decay by turning to a Liouvillian (L) description. Considering only decay, at rates *γ*_*a*_ and *γ*_*b*_ for modes *a* and *b* respectively, this describes the dynamics of particles with a lifetime (given by the inverse decay rate), starting from an initial state, e.g., following a pulsed excitation. In this regime, as already commented, one can observe a perplexing switching between the two regimes of relative phase, shown in Fig. 1(c,d). The reason for this behaviour is readily understood on the normalized Bloch sphere, where the running or oscillating phase is a topological feature of a trajectory encircling, or not, the axis of observables. The trajectory on this sphere in presence of decay is shown in Fig. 6(a). It is helical as it drifts along the 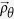 axis, from i) the initial point *P* which distance from the center on the 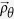 axis is given by Eq. (5), and phase by:




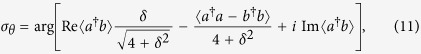




to ii) one pole of the sphere, still along the 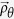 axis, depending on which particles, *a*_*θ*_ or *b*_*θ*_ have the smaller lifetime. The distance *ρ*_*θ*_(*t*) at intermediate times is given in good approximation by:




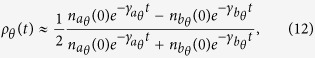




where 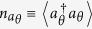 and 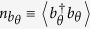 are polariton populations given in the Methods as 
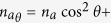

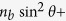

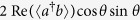
, 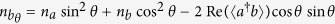 and 
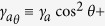

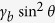
, 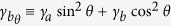. Now, in the cases where the 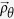 axis is not aligned with the observable 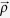 axis—which is the case out of resonance—and if the initial and final points on 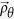 are on opposite sides of its zero, then the circle will come to encircle for some time the 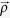 axis, corresponding to the running regime of relative phase, until it drifts again on the other side of the sphere, at which point the system goes to the oscillatory regime. It can happen that this spiral will pass by the north or south pole of the 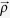 axis, which means that in the basis of observables, one population becomes exactly zero, leading to an undefined relative phase. This is not, however, compulsory. Depending on the interplay between decay and detuning, the trajectory remains the whole time on one side of the sphere, in which case the system is always in the oscillating-phase regime and there is no switching.

There are other notable behaviours that are conveniently pictured on the sphere. At resonance (*δ* = 0) and for dressed states (*ρ*_0_ = ±*N*/2), when *γ*_*a*_ = *γ*_*b*_, the relative phase starts at *π*/2 and is then locked at ±*π*/2 forever. This is a manifestation of optimal strong-coupling with full-amplitude Rabi oscillations at the Rabi frequency. Moreover, the population imbalance oscillates in time around *ρ* = 0 while decaying toward zero. Still at resonance, but now when *γ*_*a*_ ≠ *γ*_*b*_, the relative phase oscillates in time taking all the values between ±*π*/2 while the population imbalance decays faster as compared to the former case. Out of resonance, *δ* ≠ 0, when *γ*_*a*_ = *γ*_*b*_, the relative phase exhibits the same trend as in the Hamiltonian regime, however, the population decays in time.

### Classification of fixed points

The stability analysis in the Liouvillian case shows that the dynamics is richer and visits extended areas of the stability diagram. This results in the family of L points in Fig. 3 that we introduce and discuss individually below. This new phenomenology is an important consideration for polaritons that are inherently finite-lifetime particles.

The same stability treatment as before but now in presence of decay yields for *τ* and Δ:




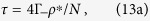







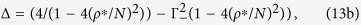




where Γ_−_ ≡ *γ*_*a*_ − *γ*_*b*_ gives access to new types of fixed points in the Δ > 0 region, namely, the system can also spiral towards its fixed points (LUD and 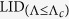 points), as expected from decay, instead of always orbiting them as before (LUR and 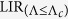 points). An example of a LUD trajectory, i.e., in the non-interacting (Rabi) regime and in presence of detuning and decay, is given in Fig. 6.

From the layout of points in Fig. 3, the stability property of the fixed points in presence of decay thus remains a good criterion to set apart the Rabi and Josephson regimes. Λ_*c*_ is still defined according to Eq. (10), but becomes time-dependent when *v*_*a*_ ≠ *v*_*b*_ since in this case it depends through Δ*E* (Eq. (3a)) on the total population *N* that decays essentially like *N*(*t*) ≈ *N*(0)exp(−(*γ*_*a*_ + *γ*_*b*_)*t*/2) (the exact solution is more complex due to interactions and may exhibit complicated patterns with abrupt variations in some particular cases, with a dynamics that would deserve an analysis of its own). The dependence of Λ_*c*_ as function of time and the detuning in interactions, *v*_*a*_ − *v*_*b*_, is shown in Fig. 5(b) for the case of bare-mode resonances, *δ* = 0, where it is seen that *v*_*a*_ = *v*_*b*_ makes it time-independent indeed and pinned to the textbook value Λ_*c*_ = 2, while an interaction imbalance results in a dependence of Λ_*c*_ similar to that due to detuning (cf. Fig. 5(a)). That is, the threshold for the Josephson regime is increased and decays in time down to the value Λ_*c*_ of Eq. (10) at long times. The Rabi regime is therefore always recovered since also Λ, Eq. (3b), decays with time, proportionally to *N*.

We now briefly discuss the various fixed points of the dissipative case that appear in Fig. 3. Although with decay only (no pumping), the steady state is the vacuum, it is approached in a limit that is well-defined and that allows a nontrivial discussion of the fixed points that remain clearly identified on the normalized Bloch sphere. This shows again the value of this ghost object, now of varying radius, that supports the dynamics of relative phase and population imbalance in a transparent way and provides insights even for the vacuum. To distinguish this variation of a dynamically evolving sphere from the conventional Bloch sphere, we would propose a dedicated terminology and refer to it as a “Paria sphere” (after the American ghost city).

#### Non-interacting case

In the dissipative Rabi regime, that is, with decay but no interactions, the fixed points are given by:




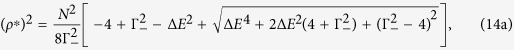







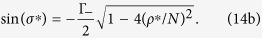




Therefore, for zero detuning and |Γ_−_| ≤ 2, one finds the fixed points at 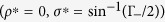 and even in the dissipative regime, these fixed points remain centers (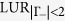). Increasing |Γ_−_| makes two consecutive centers from the set of fixed points approach each other along the *ρ* = 0 axis until they meet when |Γ_−_| = 2 with the common phase *σ* = (2*k* + 1)*π*/2 for integer *k*, at which point they become degenerate, as the 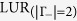 points. For |Γ_−_| > 2, the fixed points split again but now along the *σ* axis, as they keep a common value for the phase but depart in population imbalance according to 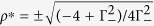. Past |Γ_−_| > 2, the fixed points also change their stability property to become spiral points (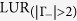). Beside, they are now connected by streamlines in the (*ρ*, *σ*) space, i.e., starting close from the unstable point brings the system towards the other point, that is stable. At non-zero detuning, the fixed points always are of the spiraling type, LUD. Finally, it can be shown that the condition *τ*^2^ − 4Δ < 0 separating spirals from nodal points is always satisfied, so the system is at most spiraling.

#### Interacting case

**Figure 7:**
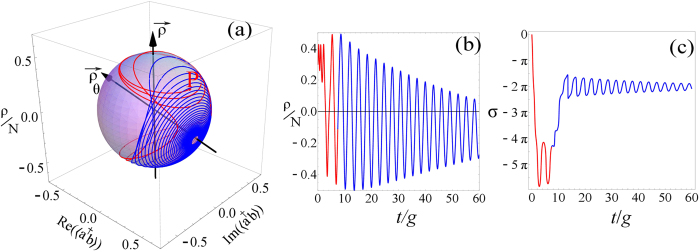
(**a**) Switching from the Josephson (red trace) to the Rabi (blue trace) regimes in an interacting, dissipative system. Here we set *va* = *vb*. P is the starting point in a system of the 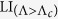 type. Due to decay, the system eventually switches to the Rabi regime, with the dynamics ending at a point on the *ρ*_*θ*_ axes. (**b**) The population imbalance shows two kinds of self-trapping, one at early times (in red) that is induced by the interactions, the other at later times (in blue) that is induced by detuning. (**c**) The relative phase also exhibit a switching between the oscillating- and running-phases, in a way such that all the four possible combinations (Josephson–Running-phase; Josephson–Oscillating-phase; Rabi–Running-phase and Rabi–Oscillating-phase) happen in succession. Parameters: *ρ*_0_ = 0.3 *N*, *σ*_0_ = 0, Δ*E* = 0.5, Λ(0) = 12, *γ*_*a*_ = 0.25 *g* and *γ*_*b*_ = 0.05 *g*.

Clearly, with decay, the total number of particles decays with time, and even if starting in the Josephson regime, ultimately the system gets into the Rabi regime where tunneling (or coupling) dominates over the interactions. That is, the system eventually follows the linear dynamics and admits the same fixed points as in the previous section (Eqs (14)). Such a transition between two regimes might in fact be the clearest experimental evidence of the Josephson regime in a dissipative context. Even though both may qualitatively appear similar, their juxtaposition in time should clearly evidence the transition from the Josephson to the Rabi regime. This is illustrated in Fig. 7, that starts from a point well in the Josephson regime, i.e., a 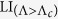 point in Fig. 3. Then Λ decays along with the number of particles as time passes, and the helix drifts till Λ = Λ_*c*_ at which point the dynamics switches to the Rabi regime (now plotted in Blue as compared to Red in the Josephson regime), and subsequently spirals along the *ρ*_*θ*_ axis. Such a switching gives rise to two kinds of “population trapping”, i.e., nonzero time-averaged population imbalance 〈*ρ*〉. One trapping is caused by the interactions, and occurs in the Josephson regime, while another type of trapping is caused instead merely by detuning, and occurs in the Rabi regime. Just as the distinction between the Rabi and the Josephson regimes might be arduous to make in cases where interactions, detuning and decay compete, also the type of trapping could be ambiguous. A decay-induced switching of regime is shown in Fig. 7(b), with two types of trapping on both sides of the switching. Figure 7(c) shows the behavior of the relative phase versus time, changing from running-phase to oscillatory as Λ decays, up to the switching time, after which point the phase runs again but now in the Rabi regime until, eventually, it is brought back to the oscillating-phase regime (still in the Rabi regime). With this example, one can get a hint at the diversity of the possible regimes, both for the dynamics of the relative phase (running and oscillatory) and for the type of the oscillations (Josephson and Rabi). While the interaction mediates the change of regime, decay mediates the change in the phase dynamics. In total, we have four combinations that succeed to each others, that illustrate well the complexity of the phenomenon when considered in its full generality. So far, such a characteristic phenomenology that would unambiguously demonstrate the Josephson regime in a strongly dissipative system has not been observed experimentally (cf. ref. 29 that is the most advanced work to date). Beside, such a representative case of the general situation of dissipative Josephson dynamics does not either exhaust the possible phenomenology. For instance, in some configurations of *v*_*a*_ ≠ *v*_*b*_, *ρ* > 0 and *δ* > 0, one can get the opposite counter-intuitive scenario where the system starts in the Rabi regime and makes an incursion later in time into the Josephson dynamics due to the interplay of both time-dependent Λ and Λ_*c*_. It is also possible to go through a double sequence of Josephson and Rabi regimes. Overall, the combination of decay and nonlinearity for particles with different interactions thus makes their dynamics considerably richer than has been contemplated before.

## Conclusions

In conclusion, we have generalized and unified the problem of Rabi and Josephson oscillations between two weakly interacting condensates to include i) detuning, ii) different interactions for each condensate and iii) decay. Our results show that even at the simplest mean-field level, such a fundamental problem had kept some important features hidden through the particular cases that had been focused on so far. For instance, the behaviour of the relative phase *σ* and population imbalance *ρ* that are usually regarded as the defining indicators of the Josephson dynamics can in fact also exhibit a running phase and population trapping in the pure Rabi regime. We have shown how their qualitative behaviour depends on a choice of representation, that is elegantly captured on a Bloch sphere of varying radius (that we termed a Paria sphere) and that clarifies an otherwise perplexing dynamics such as a change of regime of the relative-phase from oscillating to running and oscillating again for particles with a finite lifetime. An unambiguous general criterion to identify the Rabi (Λ < Λ_*c*_) and Josephson (Λ > Λ_*c*_) regimes has been provided through the critical effective interaction Λ_*c*_, Eq. (10), that generalizes the case found in the literature at resonance and for equal interactions, in which case Λ_*c*_ = 2. Although detuning can result in a Josephson-looking phenomenology, it actually makes this regime more difficult to reach, especially when caused by different interactions for the modes: *v*_*a*_ ≠ *v*_*b*_. In the Hamiltonian case, when Λ < Λ_*c*_, there are two fixed points that are centers for the dynamics. When Λ > Λ_*c*_, there four fixed points, the one at *σ* = 0 and lying between the two other fixed points being a saddle point, with all other points being centers. Similar analyses have been undertaken in the Liouvillian case and are summarized in Fig. 3. Since in this case the number of particles decays in time, the system always eventually gets into the Rabi regime. In the case of different interaction strengths, also the critical Λ_*c*_ becomes time dependent, which can lead to alternating regimes beyond the expected Josephson to Rabi transition. Rather than the observation of mere oscillations and/or population trapping, a clear identification of the Josephson regime for finite-lifetime particles can instead be made by observing the transition in time from one regime to the other. These results have relevance for all Bosonic Josephson Junction sytems but becomes pivotal for polaritons, that are strongly affected by most of the new parameters of our general description.

## Methods

We address the problem through conventional mean-field methods to integrate the Hamiltonian Eq. (1), where *a* and *b* are ground state annihilation operators and the averages 〈*a*〉 and 〈*b*〉 are order parameters (*c*-numbers) for the two condensates. Namely, we assume that the states remain coherent, which implies 〈*a*^†^*bb*^†^*b*〉 = 〈*a*^†^*b*〉〈*b*^†^*b*〉 and 〈*a*^†^*ba*^†^*a*〉 = 〈*a*^†^*b*〉〈*a*^†^*a*〉 and leads to Eq. (2). In presence of decay, the system is described by a Liouville–von Neuman equation^53^,^54^:




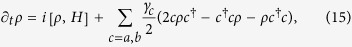




where *γ*_*c*_ are decay rates for the states *c* = *a*, *b*. Equation (2) in this dissipative regime become:




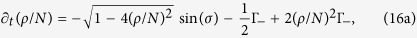







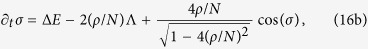




where Γ_±_ ≡ (*γ*_*a*_ ± *γ*_*b*_)/2. Equations (2) and (16) are the traditional form for the (bosonic) Josephson dynamics, coupling population imbalance and relative phase in a way that supports the notion that one drives the other. This form conceals, however, the more fundamental structure that underpins the relationship between the key variables: population imbalance indeed, but the full complex correlator 〈*a*^†^*b*〉 rather than merely its phase (once the connection is understood, however, one can indeed limit to the phase). The trajectories can be obtained from the full set of equations, more common in the quantum optical community:




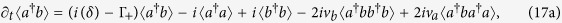







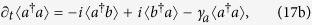







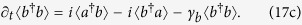




Diagonalizing these equations, we get one key result for the dynamics, Eq. (4), that states that the dynamics evolves on a sphere. This result holds even in the interacting case (with *v*_*a*_ ≠ 0 and/or *v*_*b*_ ≠ 0), but since the Hamiltonian then needs be diagonalized at all times, this is mainly a formal way to rewrite the equation. In absence of interactions, the dynamics is simply that of circles on a sphere. Here, the main, albeit obvious, argument is the introduction of the generic equation for *θ*, the *mixing angle* between exciton and photons, describing a change of basis:




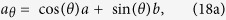







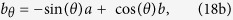




where 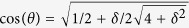. Such a transformation also points at a sphere to capture the geometry of the problem, with the exciton and photon amplitudes providing a parametrization of a Bloch vector **v**^40^:




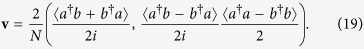




The typical representation in a (*ρ*, *σ*) plane^19^ produces instead complex patterns, even in the linear case of simple circular motion, as a result of the transformation involved by projecting from a sphere. The pure Rabi regime admits closed-form solutions, namely, for the population imbalance *ρ*:




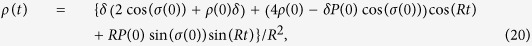




where we introduced 
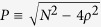
 and 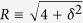; and for the relative phase *σ*(*t*) (it can also be obtained from the real and imaginary parts of *n*_*ab*_ = 〈*a*^†^*b*〉):




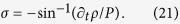




This is an exact, albeit obscure, description of the dynamics that is put in full view geometrically on the Bloch sphere. Indeed, the comparison between the particular case Eqs (20, 21) with the general solution Eq. (4) shows the great simplification brought by the geometric representation. In recent years, it has however become more common to represent the Josephson dynamics on its appropriate geometry^50^,^55^,^56^. From Eqs (20, 21), one can derive the conditions for oscillating or running phase in the Rabi regime, by considering whether *σ*(*t*) is bounded in time, in which case the function is oscillating. This is achieved by finding zeros for its derivatives, leading to the following equation for the frontier between the two regimes of phase dynamics as a function of detuning and initial conditions:




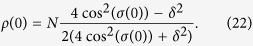




If *ρ*(0) is less than the rhs, then the phase is oscillating, otherwise it is running. The nonlinear case has no closed-form solution to the best of our knowledge although as a two-dimensional dynamical system, its solution are readily obtained numerically. We provide separately an applet to compute the trajectories on both the sphere and projected on the phase-space^57^.

## Additional Information

**How to cite this article**: Rahmani, A. and Laussy, F. P. Polaritonic Rabi and Josephson Oscillations. *Sci. Rep.* **6**, 28930; doi: 10.1038/srep28930 (2016).

## Supplementary Material

Supplementary Information
