# Supplementary material for: Polaritonic Rabi and Josephson Oscillations
Source: Sci Rep. 2016 Jul 25;6:28930. doi: 10.1038/srep28930 (PMC4958973; doi:10.1038/srep28930)
Supplement: Supplementary Information [file srep28930-s1.pdf]

Polaritonic Rabi and Josephson Oscillations

Supplementary Material

A.Rahmani[1], and F.P. Laussy[2,3,*]
[1] Physics Department, Yazd University, P.O. Box 89195-741, Yazd, Iran
[2]Condensed Matter Physics Center (IFIMAC), Universidad Autónoma de Madrid, Spain
[3]Russian Quantum Center, Moscow, Russia
*fabrice.laussy@gmail.com

Introduction

In this Supplementary Material, we briefly overview the case where incoherent pumping and upper-polariton lifetime are also (independently) present. The problem cannot be exhausted in a single study, and we provide these further analyses mainly to illustrate to which depth the dynamics of two coupled oscillators can be pursued along the lines of the main text. As far as pumping is concerned, two other works are also useful in this perspective: Gerace *et al.* have considered Josephson effects in the steady state of coherently driven cavity QED systems and tranposed its dynamics to the autocorrelations [1]. We will use a similar approach. Voronova *et al.* have reported peculiar instabilities akin to Kapitza's pendulum in driven interacting Rabi–Josephson systems [2]. We will assume here the simplest type of incoherent pumping and ignore interactions (or absorb them in an effective detuning) and we refer to Ref. [2] for an example with a more elaborate model of pumping (through a reservoir) and interactions, hinting at the complexity of the more general cases. Finally, we also discuss possible experimental observations when only one field is accessible, which is the case for instance with the internal type of Josephson oscillations.

Incoherent Pumping

With incoherent pumping, the Liouvillian acquires a new term:

$$\partial_t \varrho = i[\varrho, H] + \sum_{c=a,b} \frac{\gamma_c}{2}(2c\varrho c^\dagger - c^\dagger c\varrho - \varrho c^\dagger c) + \sum_{c=a,b} \frac{p_c}{2}(2c\varrho c^\dagger - c^\dagger c\varrho - \varrho c^\dagger c)\,, \tag{S1}$$

with p_c the incoherent rate of excitation of mode $c = a, b$. The effective decay rates are then:

$$\Gamma_\pm \equiv (\gamma_a - p_a \pm (\gamma_b - p_b))/2\,, \tag{S2}$$

which is the only change to the equations of motions for ρ and σ, that remain Eqs. (16) of the main text, while Eqs. (17b–c) get replaced by:

$$\partial_t\langle a^\dagger a\rangle = -\,i\langle a^\dagger b\rangle + i\langle b^\dagger a\rangle + (p_a - \gamma_a)\langle a^\dagger a\rangle + p_a\,, \tag{S3a}$$

$$\partial_t\langle b^\dagger b\rangle = i\langle a^\dagger b\rangle - i\langle b^\dagger a\rangle + (p_b - \gamma_b)\langle b^\dagger b\rangle + p_b\,, \tag{S3b}$$

whose solution gets upgraded to (cf. Eq. (4)):

$$|\langle a_\theta^\dagger b_\theta\rangle|^2 + \rho_\theta^2 = (N(t)/2)^2 + \mathcal{P}(t)\,, \tag{S4}$$

with the new term given by:

$$\mathcal{P}(t) = -\exp(-2\Gamma_+ t)\int_0^t \exp(2\Gamma_+ t')(p_a n_b + p_b n_a)dt' \,. \tag{S5}$$

Here as well, the result holds even in the interacting case with the same complication that the Hamiltonian then needs be diagonalized at all times. The solution remains otherwise essentially the same, namely, a trajectory (in the non-interacting case, an exact circle) on a sphere of varying radius: The "Paria sphere". While this seems to add little complexity, we proceed to show that further inspection reveals to be otherwise.

Dynamics of the relative phase

We consider as an illustration the dynamics of relative phase from Eqs. (17–a) and (S3) with nonzero p_a and p_b. The general case would bring us too far astray and we therefore limit our discussion to the pure Rabi regime (non-interacting case). We first consider the transient, starting from the vacuum and then briefly describe the steady state situation before giving it full attention in next section.

Starting from vacuum, the relative phase is ill-defined at $t = 0$. With pumping, populations in both states increase and a relative phase is established. Note that while $S = 0$ at all times since both $\langle a\rangle$ and $\langle b\rangle$ remain zero under incoherent pumping—that randomizes the phase of each mode—the cross-correlator $\sigma = \langle a^\dagger b\rangle$ is well defined and gets interconnected to particles tunneling in a way similar to the Hamiltonian case. This supports the idea that there is no absolute phase for the condensate but a degree of cross-coherence, or correlation, which, for the sake of convenience, we can still refer to as a relative phase (its argument, σ is for all purposes a relative phase). This phase sets itself at the value $\pm\pi/2$ at early times depending on the ratio p_a/p_b. Fig. S1 (a-b) shows its subsequent evolution along with the population imbalance. When $p_a/p_b \leq 1$, the relative phase starts from $\approx \pi/2$ and then evolves oscillatory in time towards its steady value of $\approx \pi$. Meanwhile, the population imbalance remains negative while its absolute value increases. When $p_a/p_b > 1$, the relative phase starts from $\approx -\pi/2$. Also, its time evolution is different than the case $p_a/p_b \leq 1$. As shown in Fig. S1 (a-b) for $p_a = 0.018g$, there are oscillations around zero followed by a jump to oscillations around π. This behavior is connected to the population imbalance that changes sign (from positive to negative), crossing zero. For the oscillations in the relative phase to end up with its steady state around 0, the population imbalance must remain positive. At resonance ($\delta = 0$), the relative phase remains $\pm\pi/2$ for any pumping ratio, and the population imbalance shows damped oscillations around zero. This is shown in Fig. S1 (c).

Panels (d-f) of Fig. S1 shows the corresponding dynamics on the Bloch sphere for three pumping ratios. The trajectory in each case starts from the point close to the south or north pole of the observable axis, then drifts toward a steady point near the eigenstate axis. As can be seen in the figures, the trajectory is immediately brought from the observable axis to the eigenstate one, and thus has no chance to loop around or intersect the $\vec{\rho}$ axis. As a consequence, the relative phase only displays damped oscillations. Consequently, the running-phase regime is suppressed by incoherent pumping. Finally, we have considered non-interacting systems only but also a fairly simple model of pumping, through Lindblad operators that are the direct counterpart of spontaneous decay.

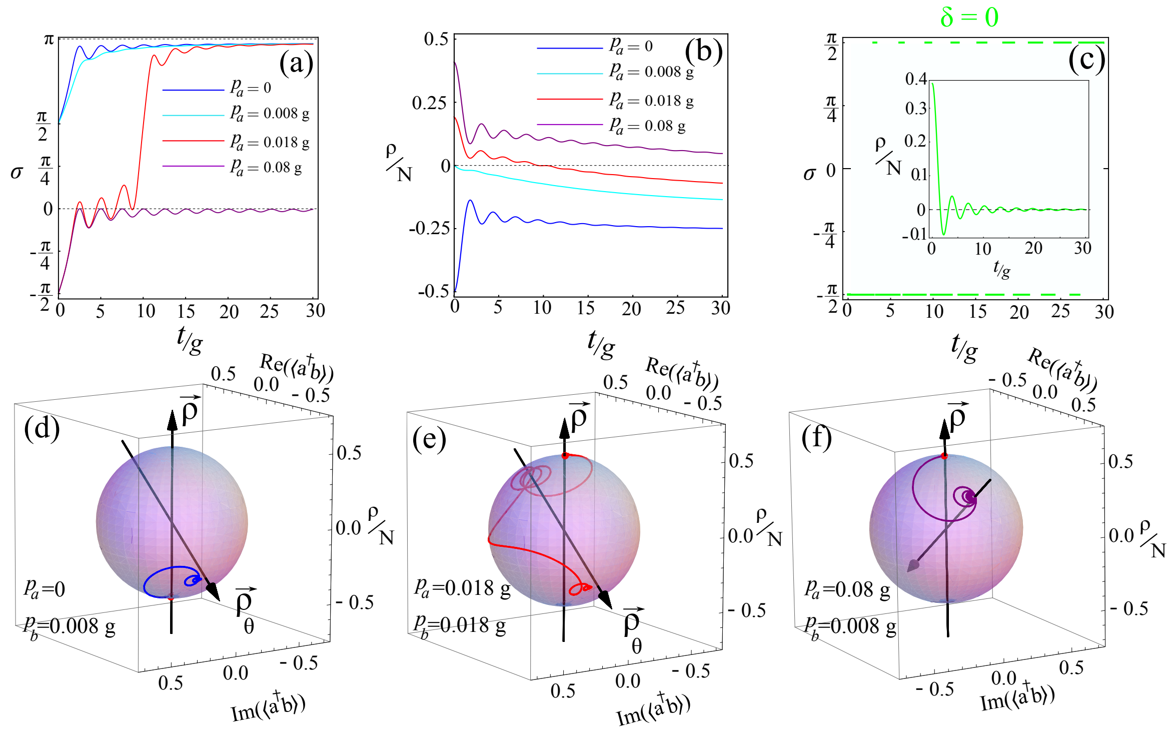

Figure S1: (a) Relative phase and (b) population imbalance in a dissipative system with pumping but in absence of interactions. Parameters: $\delta = 1.5$ and $p_b = 8 \times 10^{-3} g$. The relative phase only display the oscillatory regime even though it can jump between fixed points. (c) Relative phase at resonance ($\delta = 0$), in which case, for any pumping ratio, it is a two-valued sawtooth function. In this case, the population imbalance goes toward zero. (d–f) Dynamics on the Bloch sphere for the three different pumping ratio considered in panel (a). The trajectories never encircle the vertical $\vec{\rho}$ axis, thereby suppressing the running-regime of the phase. Parameters for all panels: $\gamma_a = 0.2g$ and $\gamma_b = 0.02g$.

Classification of fixed points

The stability analysis in presence of pumping brings some qualitative novelties due to the randomization of the phase. First, the fixed points now lie in a four-dimensional space instead of two before, since in the steady state the phase acquires a definite complex value, adding two dimensions and making obsolete the criterion of running vs oscillating phase on the Paria (normalized Bloch) sphere. Solving Eqs. (7) in the steady state (with $v_a = v_b = 0$), one finds:

$$n^* = [p_a(4\Gamma_+ - (\Gamma_- - \Gamma_+)(\delta^2 + \Gamma_+^2)) + p_b(4\Gamma_+ + (\Gamma_- + \Gamma_+)(\delta^2 + \Gamma_+^2))]/Y\,, \tag{S6a}$$

$$\rho^* = -[(\delta^2 + \Gamma_+^2)(\Gamma_+(p_b - p_a) + \Gamma_-(p_a + p_b)]/2Y\,, \tag{S6b}$$

$$\mathrm{Re}[\langle a^\dagger b\rangle^*] = \delta(\Gamma_+(p_b - p_a) + \Gamma_-(p_a + p_b))/Y\,, \tag{S6c}$$

$$\mathrm{Im}[\langle a^\dagger b\rangle^*] = (\Gamma_+/\delta)\mathrm{Re}[\langle a^\dagger b\rangle^*]\,, \tag{S6d}$$

where Y stands for $(\Gamma_-^2 - \Gamma_+^2)(\delta^2 + \Gamma_+^2) - 4\Gamma_+^2$. Note that Eqs. (S6c–S6d) are more simply expressed as $\sigma^* = \arctan(\Gamma_+/\delta)$. While the dimension of the space is larger, there is however a single fixed point, due to the unicity of the steady state solution. The stability of this point follows from the eigenvalues of the Jacobian matrix, these being:

$$\lambda_{pq} = -\Gamma_+ + pi\sqrt{qX + \sqrt{X^2 + 4\Gamma_-^2\delta^2}}\,, \tag{S7}$$

where $X \equiv \Gamma_-^2 - \delta^2 - 4$ and where p and q take the values ± 1 (we label them with the sign only, so that, e.g., $\lambda_{+-} = -\Gamma_+ + i\sqrt{-X + \sqrt{X^2 + 4\Gamma_-^2\delta^2}}$). The corresponding eigenvectors v_{pq} provide the directions in the (ρ, n, σ) four-dimensional space along which the system flows when slightly perturbed. There is no obvious geometrical features to characterize v_{pq}. The stability properties are the following: if $\Gamma_+ > 0$ and $Y < 0$, the fixed point is stable. If only $\Gamma_+ > 0$ is satisfied, the subspace spanned by $\{v_{--}, v_{-+}, v_{+-}\}$ is stable while any combination involving v_{++} is unstable. On the contrary, if only $Y < 0$ is satisfied, the only stable subspace is spanned by v_{++} while any other possible linear superposition yields an unstable point. For $\Gamma_+ < 0$, the dynamics is generally unstable, and, interestingly, can feature saddle-type of instability, that in the Hamiltonian or dissipative regime (without pumping), was used as a criterion for the Josephson regime, where the dynamics is ruled by the (weak) interactions. Here the system has no interactions, but can still manifest this type of saddle instability and in different ways, for instance when the condition $\Gamma_+ < 0$ and $Y < 0$ is met. The presence of a saddle-type of instability in a non-interacting system may be disconcerting, because this served in the previous cases as a robust criterion to identify the Josephson regime, so arguably doubts may arise on what precisely defines the Josephson regime in the most general situation. One could then look for a deeper characterization to establish such a general criterion when also including pumping, for instance, through the number of fixed points, or one could also upgrade the Josephson regime to the realm of pumping non-interacting systems. However, these various approaches, although they match the facts, lack a clear physical motivation, so we leave it an open question whether a general definition is suitable in the most general case that combines pumping, decay, detuning and interactions. Another case worthy of interest is $\Gamma_+ = 0$ and $Y = 0$, that results in pure imaginary eigenvalues. This gives rise to a center subspace, that results in a type of bifurcation known as a "transcritical bifurcation" [5], that is, the fixed point exchanges its stability when passing by $\Gamma_+ = 0$ or $Y = 0$.

Dynamics in autocorrelations

We have clarified the Rabi and Josephson dynamics in real time thanks to a normalized Bloch sphere and articulated the fundamental links between the phase and population imbalance in both cases of a pure Hamiltonian dynamics and, above, in the transients of a driven dissipative system. In this section, we explore possible similar relationships in a steady state situation, where the t dynamics has converged to constant values by definition, with only dynamics in the correlations remaining. The natural operators to consider are:

$$G_{ab}^{(1)}(\tau) = \lim_{t\to\infty} \langle a^\dagger(t+\tau) b(\tau)\rangle\,, \tag{S8a}$$

$$G_{ab}^{(2)}(\tau) = \lim_{t\to\infty} \langle a^\dagger(t) b^\dagger(\tau+t) b(\tau+t) a(t)\rangle\,. \tag{S8b}$$

The former, the crossed first order correlation function $G_{ab}^1(\tau)$, is related to coherence between the states and suggests as an extension for steady states of σ, the relative phase in autocorrelation time $\varphi \equiv \arg(G_{ab}^{(1)})$. The latter is related to fluctuations of the population imbalance in autocorrelation time. Correspondingly, the question pauses itself whether these two observables are geometrically connected or not. Using Eqs. (7) and the quantum

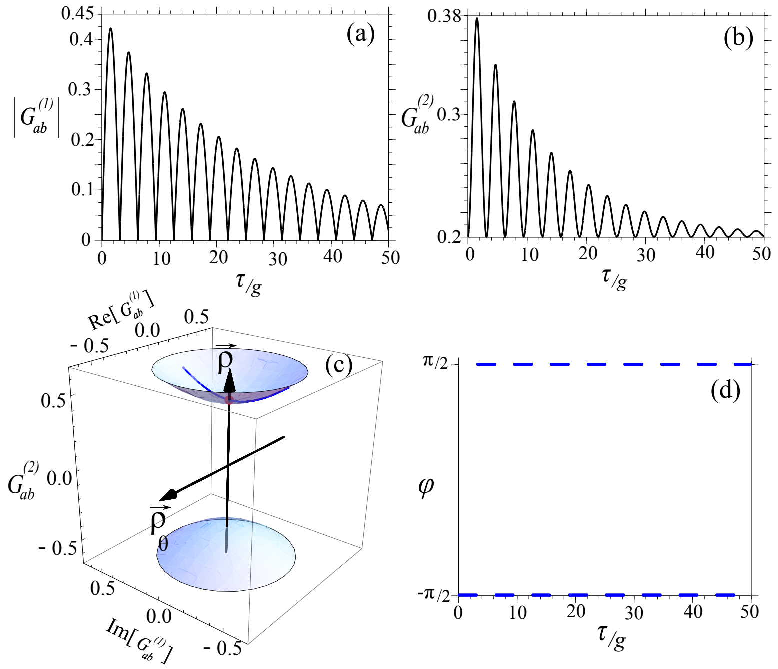

Figure S2: (a–b) τ–Dynamics of the two-time correlators $G_{ab}^{(1)}$ and $G_{ab}^{(2)}$ in the steady state of a dissipative system at resonance and without interactions. (c) In autocorrelation time, rather than a sphere, the trajectory lies on the upper sheet of an hyperboloid. A red point indicates the starting point. (d) Phase of $G_{ab}^{(1)}$, in the oscillating-regime.

regression theorem, the following relationship can be obtained:

$$G_{ab}^{(1)}(\tau) = \lim_{t\to\infty} \frac{1}{\omega} \exp((\delta - i\Gamma_+)\tau/2)$$
$$\left[\langle a^\dagger(t)b(t)\rangle(\omega\cos(\omega\tau/2) + (i\delta - \Gamma_-)\sin(\omega\tau/2)) - 2i\langle a^\dagger(t)a(t)\rangle \sin(\omega\tau/2)\right], \quad \text{(S9)}$$

where we have introduced a complex frequency $\omega = \sqrt{4 + (\delta - i\Gamma_-)^2}$. By also writing the equation of motion for $G_{ab}^{(2)}(\tau)$, it can be shown that Eqs. (S8a-S8b) are related through the relation:

$$G_{ab}^{(2)}(\tau) = |G_{ab}^{(1)}(\tau)|^2 + \lim_{t\to\infty} \frac{N^2}{4}\,. \quad \text{(S10)}$$

This shows that $G_{ab}^{(1)}$ and $G_{ab}^{(2)}$ are coupled indeed, with a possible similar Josephson interpretation that one is driving the other. However, their connection is through a two-sheet hyperboloid and is thus completely different than the the dynamics of real-time observables, even for the transient dynamics, where variables are connected via a sphere of variable radius (the Paria sphere).

Fig. S2 shows the trajectory in the hyperboloid at resonance ($\delta = 0$). In this case, the relative phase is a two-valued function, oscillating between $\pm\pi/2$ (Fig. S2 (d)), which corresponds to the the oscillatory regime of the relative phase. Correspondingly, $G_{ab}^{(1)}$ and $G_{ab}^{(2)}$ oscillate in time with a decay toward zero for $G_{ab}^{(1)}$ and $N^2/4$ for $G_{ab}^{(2)}$. Comparing with panel (d), it is observed that φ changes value whenever $G_{ab}^{(1)}$ becomes zero. This shows a similar behaviour than in the real-time behaviour where whenever the relative phase becomes ill-defined due to one state becomings zero avoid, it changes its regime. Here, ill-defined coherence changes the value of phase instead. Simultaneously, $G_{ab}^{(2)}$ reaches $N^2/4$, which is the point of lowest possible fluctuations. The corresponding trajectory on the hyperboloid

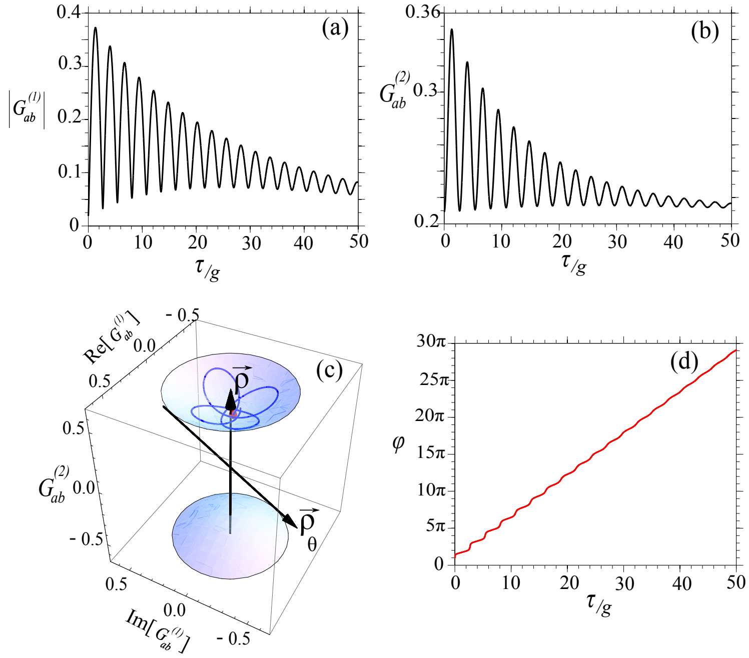

Figure S3: Corresponding τ–Dynamics of $G_{ab}^{(1)}$ and $G_{ab}^{(2)}$ of Figure S2 but in a detuned system. (c) The dynamics on the hyperboloid is now open and as a result, (d), the the phase of $G_{ab}^{(1)}$ is in the running-regime.

in panel (c) shows a simple line (in the curved space) with $\mathrm{Re}[G_{ab}^{(1)}] = 0$ and $G_{ab}^{(2)}$ swinging around the lowest point of the hyperboloid. The detuned case ($\delta \neq 0$) is shown in Fig. S3. This time, as seen in panel (d), the relative phase is running. Both $G_{ab}^{(1)}$ and $G_{ab}^{(2)}$ oscillate and decay toward different steady points as compared to resonance. The trajectory on the hyperboloid shows this time an open orbit, encircling the hyperboloid $G_{ab}^{(2)}$ axis without ever touching it. In contrast to the real-time dynamics on the Paria sphere, in autocorrelation time, there is a reduced phenomenology and, in particular, no preferred change of basis, e.g., there is no counterpart of a regime of drifting phase out of resonance and two-valued $\pm\pi/2$ phase at resonance. This is due to the Hamiltonian dynamics being washed out by the incoherent pumping.

Upper-Polariton lifetime

For the internal type of Josephson dynamics, the decay in Eq. S1 is for the bare states (excitons and photons). We have shown in the main text that the features of Rabi and Josephson oscillations are well preserved in the presence of such decay channels. There are many other possibilities for dissipating polaritons, such as the exciton pure-dephasing or upper-polariton dissipation. The latter can also be in the form of a pure-dephasing—not affecting populations but turning upper polaritons into uncorrelated excitons and photons—or, like for the bare states, consists in a decay of their populations, lowering both the number of photons, of excitons and or upper polaritons but leaving lower polaritons unaffected. In the following, we will neglect pure dephasing, as it brings a new dimension to the problem that needs to be studied on its own, and we limit our discussion to such a finite lifetime. Calling $q \equiv a_\theta$ the upper polariton state for an arbitrary θ, i.e., $q = \sin(\theta)a + \cos(\theta)b$, the

upper polariton lifetime is described by a new term in the Lindblad form of the type:

$$\mathcal{L}_u = \frac{\gamma_u}{2}(2q\varrho q^\dagger - q^\dagger q\varrho - \varrho q^\dagger q)\,. \tag{S11}$$

The trajectories are now ruled by the following equations of motion:

$$\begin{aligned}
\partial_t(\rho/N) &= -\sqrt{1-4(\rho/N)^2}(\sin(\sigma) - (\rho/N)\frac{\gamma_u}{2}\sin(2\theta)\cos(\sigma)) \\
&\quad - \frac{1}{2}(\Gamma_- - \frac{\gamma_u}{2}\cos(2\theta))(1-4(\rho/N)^2)\,, &\text{(S12a)}\\
\partial_t\sigma &= \Delta E - 2(\rho/N)\Lambda + \frac{1}{\sqrt{1-4(\rho/N)^2}}(4\rho/N\cos(\sigma) + \frac{\gamma_u}{2}\sin(2\theta)\sin(\sigma))\,, &\text{(S12b)}
\end{aligned}$$

to be compared with Eqs. (2) of the text. Despite their more complicated form, they are of the same nature as the dynamics with bare-states lifetime only, and they remain on the Paria sphere. The fixed points assume a similar expression (cf. Eqs. (14) of the text):

$$\begin{aligned}
(\rho^*)^2 &= \frac{N^2}{8D_1^2}[-4 + D_1^2 - D_2^2 + \sqrt{D_2^4 + 2D_2^2(4+D_1^2) + (D_1^2-4)^2}]\,, &\text{(S13a)}\\
\sin(\sigma^*) &= -\frac{D_1}{2}\sqrt{1-4(\rho^*/N)^2}\,, &\text{(S13b)}
\end{aligned}$$

where D_1 appears as a generalized form of Γ_- and D_2 as a generalized form of ΔE, the two being admixed by a nonzero γ_u:

$$\begin{aligned}
D_1 &= \frac{4(4\Gamma_- - 2\gamma_u\cos[2\theta] + \gamma_u\Delta E\sin[2\theta])}{16+\gamma_u^2\sin^2[2\theta]}\,, &\text{(S14a)}\\
D_2 &= \frac{16\Delta E - 4\Gamma_-\gamma_u\sin[2\theta] + \gamma_u^2\sin[4\theta]}{16+\gamma_u^2\sin^2[2\theta]}\,. &\text{(S14b)}
\end{aligned}$$

One can check that when $\gamma_u = 0$, the case of the text is recovered. Similarly, the stability analysis of the fixed points takes a more complicated form. One finds for the τ and Δ parameters (cf. Eqs. (13)):

$$\tau = 4(\Gamma_- - \frac{\gamma_u}{2}\cos[2\theta])(\rho^*/N) + \gamma_u\sin[2\theta]\sqrt{1-4(\rho^*/N)^2}\sqrt{1-D_1^2(1-4(\rho^*/N)^2)}\,, \tag{S15a}$$

$$\begin{aligned}
\Delta &= \frac{[-4 + (\frac{\gamma_u}{2}\sin[2\theta])^2]D_2^2}{(\rho^*/N)^2} + 2\gamma_u\sin[2\theta]D_2(\Gamma_- - \frac{\gamma_u\cos[2\theta]}{2} - \gamma_u\sin[2\theta]D_2)\\
&\quad + 4D_1[-4(\Gamma_- - \frac{\gamma_u}{2}\cos[2\theta]) - D_1(4 - (\frac{\gamma_u}{2}\sin[2\theta])^2) + 4\gamma_u\sin[2\theta]D_2](\rho^*/N)^4\,. &\text{(S15b)}
\end{aligned}$$

These expressions, however, do not bring new qualitative features, and one has, like before, two spirals, one stable (the head point), the other unstable (the destination). Beside, the criterion to define the Josephson regime in terms of fixed points remains valid.

It is likely that pure dephasing of upper polaritons is a stronger effect than their finite lifetime. The dynamics is however even more complicated in this case and becomes qualitatively different. One can also contemplate the combination/competition of both terms. With pure dephasing, the upper polariton is turned into uncorrelated photons and excitons. This acts as a kind of internal pumping for the bare states, and destroys their coherence. As a consequence, the trajectories escape the Paria sphere to travel instead on ellipsoids. Such an analysis is left for a future work.

Observation through one field only

Finally, we discuss the manifestation of our phenomenology when one field only is observable. For two polariton condensates, that can be accessed individually and separately, the standard description through the relative phase and population can be made in a conventional way, in particular the relative phase is conveniently retrieved through the contrast in interferences of the two condensates. For the internal type of dynamics between the photon and exciton component of a single condensate, however, the situation is complicated by the difficult access to the exciton field. While it remains within reach in principle, one great asset of polaritons is the ease of their imaging through the photonic field. Therefore, we discuss the possibility to qualify the Rabi and Josephson regimes through observables of one field only. For simplicity, we limit our discussion to the Hamiltonian case (no decay). The equations for the absolute populations n_a, n_b and phases S_a, S_b of the modes a and b provide an alternative description of the dynamics (cf. Eqs. (2) and (17) of the main text):

$$\partial_t n_a = -2\sqrt{n_a n_b}\sin(S_a - S_b)\,, \quad \partial_t S_a = -(\epsilon_a + \sqrt{\frac{n_b}{n_a}}\cos(S_a - S_b) + 2v_a n_a/N)\,, \tag{S16a}$$

$$\partial_t n_b = 2\sqrt{n_a n_b}\sin(S_a - S_b)\,, \quad \partial_t S_b = -(\epsilon_b + \sqrt{\frac{n_a}{n_b}}\cos(S_a - S_b) + 2v_b n_b/N)\,, \tag{S16b}$$

with $N = n_a + n_b$ constant. The fixed points for this system, denoted with "*", are found by combining Eqs. (S16a) as:

$$S_a^* - S_b^* = m\pi \text{ for integer } m\,, \tag{S17a}$$

$$F_\pm \equiv -\delta \pm \frac{1 - 2n_a^*}{\sqrt{n_a^*(1 - n_a^*)}} - 2n_a^*(\Lambda_a + \Lambda_b) + 2\Lambda_b = 0\,, \tag{S17b}$$

$$G_\pm \equiv -\delta \pm \frac{2n_b^* - 1}{\sqrt{n_b^*(1 - n_b^*)}} + 2n_b^*(\Lambda_a + \Lambda_b) - 2\Lambda_a = 0\,. \tag{S17c}$$

where we introduced $\Lambda_{i=a,b} \equiv N v_i$ and we remind that $\delta \equiv \epsilon_a - \epsilon_b$. Comparing with the corresponding expressions for for the relative quantities, Eqs. (2) of the main text, this shows that, from a stability analysis point of view, the fixed points of one field alone can also discriminate between the Rabi and Josephson dynamics. If one can measure S_a independently (see Ref. [3] and references therein for a discussion of how to do so), then one can identify the Josephson regime merely by exhibiting a saddle fixed point, i.e., trajectories in the (n_a, S_a) phase space should be repelled around it. If, on the other hand, measuring S_a is not possible or convenient, then one can likewise determine the regime through the number of fixed points for the cavity population. They are shown in Fig. 4 for both the photon field (solid lines, accessible experimentally) and the exciton field (dashed lines, assumed not accessible). One can then excite the system in all the possible configurations of n_a and n_b (even if the exciton field is not observable, it is possible to control its value at initial times [4]) and count how many steady cavity fields are observed. If only one, then the system is in the Rabi regime. If two, it lies at the transition, if three, it is well within the Josephson regime.

Both experiments are certainly tedious as they require systematic measurements, but this shows that in principle one can identify the type of polariton dynamics only from conventional cavity-field measurements.

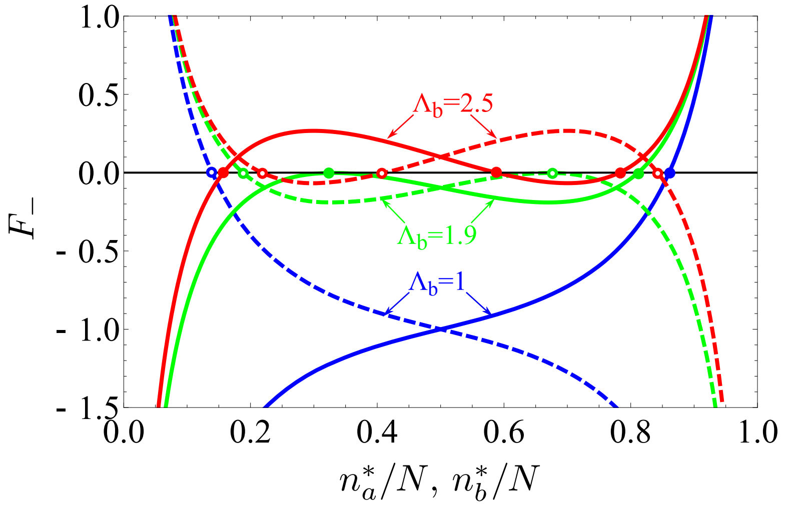

Figure S4: The fixed points in the reduced populations for the cavity field n_a^*/N (solid) and exciton field n_b^*/N (dashed) for various values of exciton interactions Λ_b are identified by circles (full/empty, respectively). Their number is in one-to-one correspondence with the number of fixed points in the (ρ, σ) phase space, cf. Fig. 4(d) of the main text, thus they can also be used as a criterion to identify the regime of the dynamics (Rabi or Josephson).

References

[1] Gerace, D., Türeci, H. E., Ĭmamoḡlu, A., Giovannetti, V. & Fazio, R. The quantum-optical josephson interferometer. *Nat. Phys.* **5**, 281 (2009).

[2] Voronova, N. S., Elistratov, A. A. & Lozovik, Y. E. Inverted pendulum state of a polariton Rabi oscillator. *arXiv:1602.01457* (2016).

[3] Voronova, N. S., Elistratov, A. A. & Lozovik, Y. E. Detuning-controlled internal oscillations in an exciton-polariton condensate. *Phys. Rev. Lett.* **115**, 186402 (2015).

[4] Dominici, L. *et al.* Ultrafast control and Rabi oscillations of polaritons. *Phys. Rev. Lett.* **113**, 226401 (2014).

[5] Strogatz, S. H. *Nonlinear Dynamics and Chaos* (Perseus Books, 1994).
